# Generating binomial coefficients in a row of Pascal's triangle from extensions of powers of eleven

**DOI:** 10.1016/j.heliyon.2022.e11651

**Published:** 2022-11-11

**Authors:** Md. Shariful Islam, Md. Robiul Islam, Md. Shorif Hossan, Md. Hasan Kibria

**Affiliations:** a Department of Mathematics, University of Dhaka, Bangladesh; b Department of Computer Science and Engineering, Green University of Bangladesh, Dhaka, Bangladesh; c Department of Applied Mathematics, University of Dhaka, Bangladesh

**Keywords:** Binomial coefficients, Pascal's triangle, Logarithm, Modular arithmetic

## Abstract

Sir Isaac Newton noticed that the values of the first five rows of Pascal's triangle are each formed by a power of 11, and claimed that subsequent rows can also be generated by a power of 11. Literally, the claim is not true for the $5^{th}$ row and onward. His genius mind might have suggested a deep relation between binomial coefficients and a power of some integer that resembles the number 11 in some form. In this study, we propose and prove a general formula to generate the values in any row of Pascal's triangle from the digits of ${\left(1\underset{\text{Θ zeros}}{\underset{︸}{0\cdots 0}}1\right)}^{n}$. It can be shown that the numbers in the cells in $n^{th}$ row of Pascal's triangle may be achieved from Θ+1 partitions of the digits of the number ${\left(1\underset{\text{Θ zeros}}{\underset{︸}{0\cdots 0}}1\right)}^{n}$, where Θ is a non-negative integer. That is, we may generate the number in the cells in a row of Pascal's triangle from a power of 11, 101, 1001, or 10001 and so on. We briefly discuss how to determine the number of zeros Θ in relation to *n*, and then empirically show that the partition really gives us binomial coefficients for several values of *n*. We provide a formula for Θ and prove that the ${\left(n+1\right)}^{th}$ row of Pascal's triangle is simply Θ+1 partitions of the digits of ${\left(1\underset{\text{Θ zeros}}{\underset{︸}{0\cdots 0}}1\right)}^{n}$ from the right.

## 1 Introduction

Algebra is a spacious part of the science of mathematics that provides the opportunity to express mathematical ideas precisely. In algebra, the binomial expansion and Pascal's triangle are considered important. Pascal's triangle is an arrangement of the binomial coefficients and one of the most known integer models. Though it was named after the French scientist Blaise Pascal, it was studied in ancient India [1], [2], Persia [3], [4], China [5], Germany, and Italy [6].

In reality, the definition of the triangle was made centuries ago. In 450 BC, an Indian mathematician named Pingala is said to have introduced the definition of this triangle in a Sanskrit poetry book. Chinese mathematicians had the same idea and named the triangle as “Yang Hui's triangle”. Later, Persian mathematician Al-Karaji and Persian astronomer-poet Omar Khayyam named the triangle as the “Khayyam triangle”. It also has multi-dimensional shapes. The three-dimensional shape is referred to as Pascal's pyramid or Pascal's tetrahedron, while the other general-shaped ones are called Pascal's simplifications.

Various studies have been conducted in many different disciplines about Pascal's triangle. For the construction of Pascal's triangle, Sgroi [7] stated that each line starts with 1 and ends with 1, and this series can be expanded with simple cross-joints. Jansson [8] developed three geometric forms related to Pascal's triangle and included examples of each form. Toschi [9] used various permutations to generate new forms of Pascal's triangles and generalized them. Duncan and Litwiller [10] addressed the reconstruction of Pascal's triangle with the individuals. Here they collected data on the opinions of individuals using qualitative methods, and determined the methods of constructing the Pascal's triangle in different ways with the attained findings.

Researchers worked on Pascal's fascinating characteristics. Using the principle of permutation, Putz [11] designed Pascal Polytope and linked it to the Fibonacci concept. Houghton [12] gave the concept of the relationship between the successive differential operations of a function and Pascal's triangle. With an application, he attempted to incorporate the idea of a differentiable function into Pascal's triangle. The relationship between Pascal's triangle and the Tower of Hanoi has been elucidated by Andreas M. Hinz [13]. Finding diagonal sum [14], k-Fibonacci sequence, recurrence relations [15], finding exponential (*e*) [16] were a part of those to describe the work that is generated from Pascal's triangle. Some fascinating properties of Pascal's triangle are available in [17], [18]. In 1956, Freund [19] elicited that the generalized Pascal's triangles of $s^{\text{th}}$ order can be constructed from the generalized binomial coefficients of order *s*. Bankier [20] gave the Freud's alternative proof. Kallós generalized Pascal's triangle from an algebraic point of view by different bases. He tried to generalize Pascal's triangle using the power of integers [21], powers of base numbers [22] and their connections with prime numbers [23]. Kuhlmann tried to generate Pascal's triangle using the T-triangle concept [24].

The concept of using a power of 11 to generate rows of Pascal's triangle was first introduced by Sir Isaac Newton. He noticed the first five rows of Pascal's triangle are formed by a power of 11 and claimed (without proof) that subsequent rows can also be generated by a power of eleven as well [25]. Arnold et al. [26] showed if one assigns a place value to each of the individual terms in a certain row of the triangle, the pattern can be seen again. Morton [27] noted the Pascal's triangle property by the power of 11 for 10 base numeral system. Mueller [28] noted that one can get the $n^{\text{th}}$ power of 11 from the $n^{\text{th}}$ row of the Pascal's triangle with positional addition.

It is clearly concluded that above mentioned works did not express a full row of Pascal's triangle from a power of 11, or from the digits of ${\left(1\underset{\text{Θ zeros}}{\underset{︸}{0\cdots 0}}1\right)}^{n}$, as Sir Isaac Newton hinted. This paper has worked on the extension from powers of 11 to powers of $101,1001,10001,\ldots (1\underset{\text{Θ zeros}}{\underset{︸}{0\cdots 0}}1)$ and proved a new general formula to generate any row of Pascal's triangle.

## 2 Methods

The very basic definition of getting the number at any cell of a row of Pascal's triangle is the summation of the numbers at the two adjacent cells of the previous row. The rows of Pascal's triangle are numbered starting from n=0 on the top and the cells in each row are numbered from k=0 on the left. For n=0, there is only one cell with the value 1. As the successive rows are generated, the numbers in the right most and left most cells are defined to be 1.

**Figure 1:**
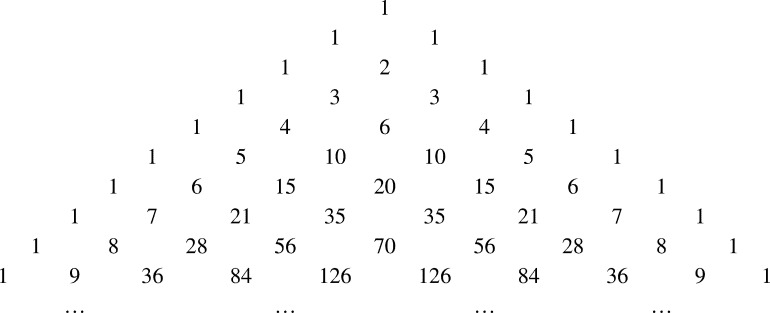
Pascal's triangle.

The power of 11 technique is generating Pascal's triangle by multiplying previous rows by 11 successively. The one digit partition of $11^{1}=11$ gives us the numbers in the cells of the $1^{\text{st}}$ row and $11^{2}=121$, $11^{3}=1331$ and $11^{4}=14641$ give $2^{\text{nd}}$, $3^{\text{rd}}$, and $4^{\text{th}}$ rows respectively. Before finding the general rule for subsequent rows, we first elaborate on the concept of powers of 11. The reason behind getting Pascal's triangle from the powers of 11 lies in the general rule of multiplication. What do we get from multiplication of a number by 11? Let $r_{n}$ be the number generated by concatenating each of the digits in the cells of the $n^{\text{th}}$ row of Pascal's triangle from left to right.

**Figure 2:**
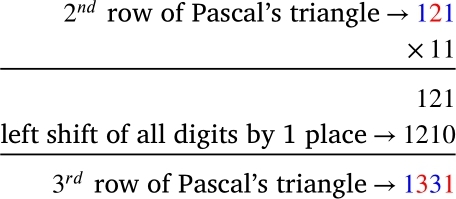
Multiplication of 2^*nd*^ row by 11.

Fig. 2 shows that multiplication of 121 by 11 gives $r_{3}$. That is $r_{3}=11r_{2}$.

**Figure 3:**
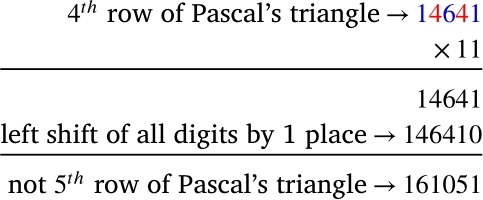
Multiplication of 4^*th*^ row by 11.

Fig. 1 indicates that $r_{5}$ should be 15101051, whereas from Fig. 3 we get 161051. So, we can make a comment from Fig. 3 that multiplication of $r_{4}$ by 11 does not give $r_{5}$.

Patently $11^{5}=161051$ and $11^{6}=1771561$, but the $5^{\text{th}}$ and $6^{\text{th}}$ row of Pascal's triangle are

**Figure:**
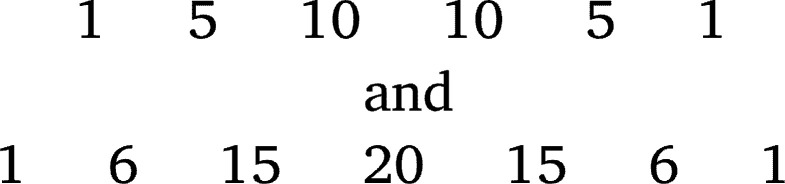

respectively. The above scheme fails for 11^5^ or 11^6^. Why does the power of 11 technique fail here, and why does the power of 11 technique work for the first four rows? If the reader closely looks at the Pascal's triangle, they will see that all of the cell values in the first to fourth rows are one digit. We get two-digit cell values for the first time in the central cells of the fifth row, which we think is a potential reason for the power of 11 technique failing here. So for finding the $5^{\text{th}}$ row onward, we need two (three, four, …) digits partitions of $r_{n}$. The shifting of places in Fig. 2 and Fig. 3 implies using a power of $1\underset{\text{Θ zeros}}{\underset{︸}{0\cdots 0}}1$, for some Θ, might work. Now, we will endeavor to formulate a specific rule.

At first, we attempt to generate the number for which two digit partitions give us the numbers in the cells of a row of the Pascal's triangle. So we extend the concept of power of 11 technique to the power of 101 technique and multiply 101 by itself to see the consequences. We can achieve this by using the very basic rules of multiplication.

**Figure 4:**
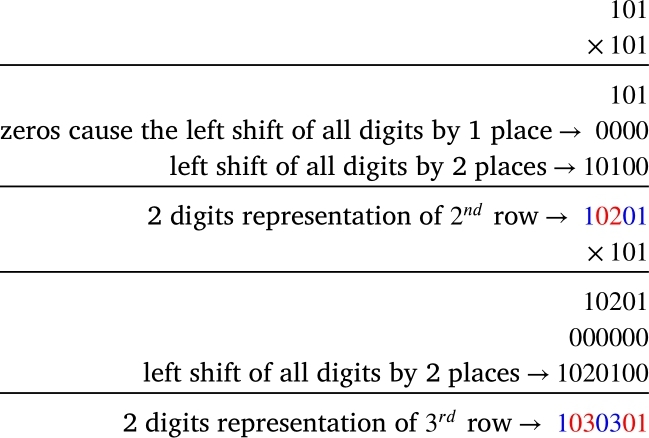
Effects of multiplying by 101.

Fig. 4 displays the impact of multiplication by 101. The result of 101×101 is 10201 whereas 11×11=121. One digit partition of 121 produce 121 but two digits partition of 10201 yields 10201 which is identical to 010201. The colored pairs of digits in each product are the summation of two numbers in the adjacent cells of the previous row.

Now, $101^{5}=10510100501$, from which we can construct $5^{\text{th}}$ row of Pascal's triangle by two digits partitions from the right.

**Figure:**
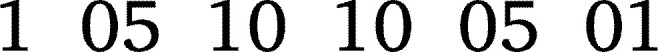

Similarly from $101^{6}=1061520150601$ and $101^{7}=107213535210701$, we can easily construct the $6^{\text{th}}$ and $7^{\text{th}}$ row respectively.

**Figure:**
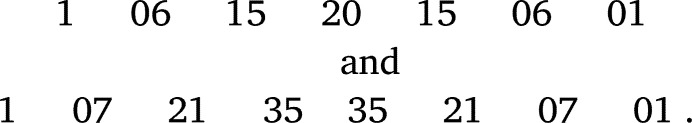

Hence, two-digit partitions of 101^5^, 101^6^ and 101^7^ generate the numbers in the cells of the $5^{th}$, $6^{th}$ and $7^{th}$ rows of Pascal's triangle, respectively, due to the insertion of one zero between . Sir Issac Newton might have meant this technique in his claim.

Can a conclusion be drawn for generating the numbers in cells of any row of Pascal's triangle with the help of some extended power of 11 technique such as $101^{n}$? The $9^{\text{th}}$ row of Pascal's triangle is

193684126126843691

Clearly, two digits partition from the right of the number $101^{9}=1093685272684360901$ does not give the numbers in the cells of the $9^{\text{th}}$ row because the numbers in the central cells of this row contains three digits.

So the representation of three place values for each entry of Pascal's triangle requires a new formula to be generated. The previous context directed that multiplication of a number by 11 and 101 makes the left shift of all digits by one and two places, respectively. Therefore, three-digit representation requires multiplication by 1001.

**Figure 5:**
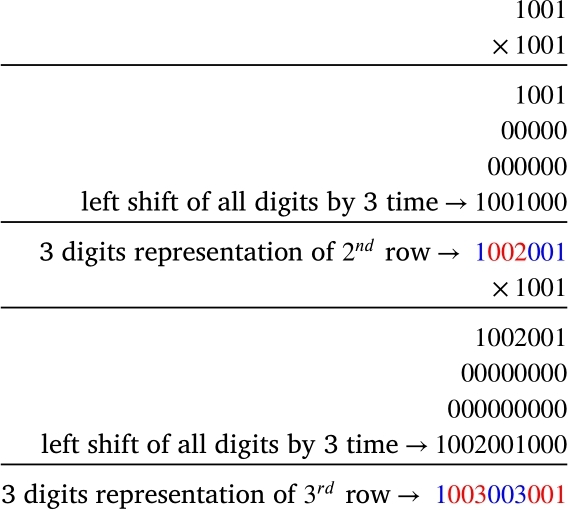
Effects of multiplying by 1001.

Fig. 5 indicates the left shift of all digits by 3 places when a number is multiplied by 1001.

By continuing the multiplication by 1001 in Fig. 4, we get

$$1001^{9}=1009036084126126084036009001$$

from which one may form the $9^{th}$ row of Pascal's triangle by three digits partition of the number from the right.

**Figure:**
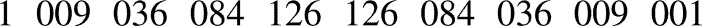

Similarly, ${\left(1001\right)}^{10}=1010045120210252210120045010001$,

1010045120210252210120045010001⟼1010045120210252210120045010001

the $10^{\text{th}}$ row of the Pascal's triangle.

From the above study, it may be concluded that the three-digit representation requires the left shift of all digits by three places, and requires two zeros between , that is 1001. Why do we require three-digit representation for the $9^{th}$ and $10^{th}$ rows of Pascal's triangle? Because the central cells of $9^{\text{th}}$ and $10^{\text{th}}$ rows are of three digits. Similarly, we need two-digit representation for $5^{th}$ to $8^{th}$ rows since the central cell of these rows are numbers of two digits. And, the first four rows are satisfied by $11^{n}$ since the central cell of the first four rows contains one digit only. So for any given row, the number of digits in the representation for the number in a cell should be equal to the number of digits in the central cell(s) of that row. Why the central cell value(s) should be taken into account in this situation may be questioned. The central cell value(s) matter here because an observation of Pascal's triangle is that for any row, the central values are the largest of any other cell values of that row. So, knowing the number of digits in the central cell value(s) implies the required number of digits in the partitioning of the representation for that particular row of Pascal's triangle.

The above discussion compels us to generate a formula to find the central values of any row of the Pascal's triangle. For an odd number, say n=9, we get n+1=10 elements in $9^{th}$ row and so the central value should be ${\left(\frac{10}{2}\right)}^{th}=5^{th}$ observation of that row, which is $\left(\begin{array}{c} 9\\ 5-1 \end{array}\right)=\left(\begin{array}{c} 9\\ 4 \end{array}\right)=126$. For an even number, say n=10, we get n+1=11 elements and the central value should be ${\left\lceil \frac{11}{2}\right\rceil }^{th}=6^{th}$ observation, which is $\left(\begin{array}{c} 10\\ 6-1 \end{array}\right)=\left(\begin{array}{c} 10\\ 5 \end{array}\right)=252$.

By taking the *floor* value of $\frac{n}{2}$, a formula for central value of $n^{th}$ row is $\left(\begin{array}{c} n\\ \left\lfloor \frac{n}{2}\right\rfloor \end{array}\right)$.

But we never need a central element; rather it is necessary to know how many digits the central element has. Applying the property of the logarithmic function, one can identify how many digits (or place values) the central element has without knowing the value of the cell. Therefore, the number of digits in the central value is given by

$$\left\lceil \log_{10}\left(\begin{array}{c} n\\ \left\lfloor \frac{n}{2}\right\rfloor \end{array}\right)\right\rceil$$

since $\left\lceil \log_{10}(X)\right\rceil$ represents the number of digits of *X* when $X\neq 10^{n}$, for n∈N. For a central value with Θ−1 digits we require Θ zeros between , such that $\left(1\underset{\text{Θ zeros}}{\underset{︸}{0\cdots 0}}1\right)$. So, the required number of zeros between  can be obtained by taking the *floor* value of $\log_{10}\left(\begin{array}{c} n\\ \left\lfloor \frac{n}{2}\right\rfloor \end{array}\right)$.

If Θ represents the number of zeros between 1 and 1 in 11, then

$$\Theta =\lfloor \log_{10}\left(\begin{array}{c} n\\ \left\lfloor \frac{n}{2}\right\rfloor \end{array}\right)\rfloor$$

We now verify it for an odd number n=9 and an even number n=10.

If, n=9 then Θ=2, and if n=10 then Θ=2.

For both of the numbers, we need 2 zeros between 1 and 1 in 11. So, to get the $9^{th}$ and $10^{th}$ rows we have to calculate 1001^9^ and 1001^10^ respectively. Both of these cases have been discussed above, and are consistent with our formula for Θ.

It's time to generate the formula to find any row of Pascal's triangle. We infer that the general formula for generating the $n^{th}$ row of Pascal's triangle is the Θ+1 digit partitioning of the digits of the number ${\left(1\underset{\text{Θ zeros}}{\underset{︸}{0\cdots 0}}1\right)}^{n}$ from the right. For n=15, we get Θ=3. So, we have to insert 3 zeros between  and the $15^{th}$ row can be constructed by four-digit partitions of the digits of the number

$$10001^{15}=1001501050455136530035005643564355005300313650455010500150001$$

from the right as shown below

1001501050455136530035005643564355005300313650455010500150001

Notice the partitioning yields the $15^{\text{th}}$ row of the Pascal's triangle

11510545513653003500564356435500530031365455105151

Similarly, we may verify for n=16, Θ=4 and

$${\left(100001\right)}^{16}=100016001200056001820043680800811440128701144008008043680182000560001200001600001$$

This $16^{\text{th}}$ row can also be verified from the existing Pascal's triangle. The above formula can be used for a large *n*. We now exemplify the $51^{st}$ row of Pascal's triangle. Hence n=51 gives Θ=14.

We have to put 14 zeros between 1 and 1 in 11, that is ${\left(1000000000000001\right)}^{51}$.



The desired $51^{\text{st}}$ row can be obtained by partitioning each 15 digits from the right. For readers' convenience, we marked each partition with different colors and showed that the above formula generates the $51^{\text{st}}$ row of the Pascal's triangle.

## 3 Results and discussion

**Remark:** In general the $r^{\text{th}}$ partition of length *k* of the digits of a positive integer *N* is the left most *k* digits of the number $\left(N\;\mathrm{mod}\;10^{r\times k}\right)$.

Now we give a proof of the power of 11 technique. To prove the main theorem, we prove some inequalities and lemmas.

For n∈N, we have the following inequalities

$$\Theta =\lfloor \log_{10}\left(\begin{array}{c} n\\ \left\lfloor \frac{n}{2}\right\rfloor \end{array}\right)\rfloor$$

From the property of floor function, $\Theta \leq \log_{10}\left(\begin{array}{c} n\\ \left\lfloor \frac{n}{2}\right\rfloor \end{array}\right)<\Theta +1$

(1) $$\Rightarrow 10^{\Theta }\leq \left(\begin{array}{c} n\\ \left\lfloor \frac{n}{2}\right\rfloor \end{array}\right)<10^{\Theta +1}\Rightarrow \left(\begin{array}{c} n\\ \left\lfloor \frac{n}{2}\right\rfloor \end{array}\right)<10^{\Theta +1}$$

Since both sides of the above inequality are integers, the difference between $10^{\Theta +1}$ and $\left(\begin{array}{c} n\\ \left\lfloor \frac{n}{2}\right\rfloor \end{array}\right)$ is at least 1, therefore

(2) $$\Rightarrow 10^{\Theta +1}-\left(\begin{array}{c} n\\ \left\lfloor \frac{n}{2}\right\rfloor \end{array}\right)\geq 1\Rightarrow 10^{\Theta +1}-1\geq \left(\begin{array}{c} n\\ \left\lfloor \frac{n}{2}\right\rfloor \end{array}\right)$$

From inequality (1), we also have

$$10^{\Theta }\leq \left(\begin{array}{c} n\\ \left\lfloor \frac{n}{2}\right\rfloor \end{array}\right)$$

For n,r∈N and 0≤r≤n, the maximum value of $\left(\begin{array}{c} n\\ r \end{array}\right)$ occurs when $r=\lfloor \frac{n}{2}\rfloor$. Hence

(3) $$\left(\begin{array}{c} n\\ \left\lfloor \frac{n}{2}\right\rfloor \end{array}\right)\geq \left(\begin{array}{c} n\\ r \end{array}\right),$$

and notice that ${\left(1\underset{\text{Θ zeros}}{\underset{︸}{0\cdots 0}}1\right)}^{n}={\left(10^{\Theta +1}+1\right)}^{n}$.

Lemma 1 *If* n,r∈N *and* 0≤r≤n *, then*

$$\left(\begin{array}{c} n\\ n-(r-1) \end{array}\right)10^{\left(r-1)(\Theta +1\right)}+\left(\begin{array}{c} n\\ n-(r-2) \end{array}\right)10^{\left(r-2)(\Theta +1\right)}+\cdots +1<10^{r(\Theta +1)}.$$

Proof By inequality (3), we have

(4) $$\left(\begin{array}{c} n\\ n-(r-1) \end{array}\right)10^{\left(r-1)(\Theta +1\right)}+\left(\begin{array}{c} n\\ n-(r-2) \end{array}\right)10^{\left(r-2)(\Theta +1\right)}+\cdots +1\;\;\;\leq \left(\begin{array}{c} n\\ \left\lfloor \frac{n}{2}\right\rfloor \end{array}\right)10^{\left(r-1)(\Theta +1\right)}+\left(\begin{array}{c} n\\ \left\lfloor \frac{n}{2}\right\rfloor \end{array}\right)10^{\left(r-2)(\Theta +1\right)}+\cdots +\left(\begin{array}{c} n\\ \left\lfloor \frac{n}{2}\right\rfloor \end{array}\right)\;\;=\left(\begin{array}{c} n\\ \left\lfloor \frac{n}{2}\right\rfloor \end{array}\right)\left(10^{\left(r-1)(\Theta +1\right)}+10^{\left(r-2)(\Theta +1\right)}+\cdots +1\right)$$

Since, $10^{\left(r-1)(\Theta +1\right)}+10^{\left(r-2)(\Theta +1\right)}+\cdots +1$ is a geometric series of *r* terms with common ratio $10^{\left(\Theta +1\right)}$,

(5) $$\sum_{i=0}^{r-1}10^{(\Theta +1)i}=1+10^{\left(\Theta +1\right)}+\cdots +10^{\left(r-1)(\Theta +1\right)}=\frac{10^{r(\Theta +1)}-1}{10^{\left(\Theta +1\right)}-1}$$

From inequality (4) and equation (5) we have,

(6) $$\left(\begin{array}{c} n\\ n-(r-1) \end{array}\right)10^{\left(r-1)(\Theta +1\right)}+\left(\begin{array}{c} n\\ n-(r-2) \end{array}\right)10^{\left(r-2)(\Theta +1\right)}+\cdots +1\leq \left(\begin{array}{c} n\\ \left\lfloor \frac{n}{2}\right\rfloor \end{array}\right)\;\frac{10^{r(\Theta +1)}-1}{10^{\left(\Theta +1\right)}-1}<\left(\begin{array}{c} n\\ \left\lfloor \frac{n}{2}\right\rfloor \end{array}\right)\;\frac{10^{r(\Theta +1)}}{10^{\left(\Theta +1\right)}-1}$$

From inequality (2), we have

(7) $$\left(\begin{array}{c} n\\ \left\lfloor \frac{n}{2}\right\rfloor \end{array}\right)\;\frac{10^{r(\Theta +1)}}{10^{\left(\Theta +1\right)}-1}\leq (10^{\left(\Theta +1\right)}-1)\;\frac{10^{r(\Theta +1)}}{10^{\left(\Theta +1\right)}-1}=10^{r(\Theta +1)}$$

From inequalities (6) and (7), we have

$$\left(\begin{array}{c} n\\ n-(r-1) \end{array}\right)10^{\left(r-1)(\Theta +1\right)}+\left(\begin{array}{c} n\\ n-(r-2) \end{array}\right)10^{\left(r-2)(\Theta +1\right)}+\cdots +1<10^{r(\Theta +1)}.$$

 □

Proposition 1 *If* n,r∈N *and* 0≤r≤n *, then*

$${\left(10^{\Theta +1}+1\right)}^{n}\;\mathrm{mod}\;10^{r(\Theta +1)}=\left(\begin{array}{c} n\\ n-(r-1) \end{array}\right)10^{\left(r-1)(\Theta +1\right)}+\left(\begin{array}{c} n\\ n-(r-2) \end{array}\right)10^{\left(r-2)(\Theta +1\right)}+\cdots +1$$

Proof Expanding ${\left(10^{\Theta +1}+1\right)}^{n}$ by binomial theorem, we have

$${\left(10^{\Theta +1}+1\right)}^{n}\;\mathrm{mod}\;10^{r(\Theta +1)}=\sum_{r=0}^{n}\;\left(\begin{array}{c} n\\ n-r \end{array}\right)\;10^{\left(\Theta +1)\{n-(n-r)\right\}}\;\mathrm{mod}\;10^{r(\Theta +1)}=\left(\begin{array}{c} n\\ n-(r-1) \end{array}\right)10^{\left(r-1)(\Theta +1\right)}+\left(\begin{array}{c} n\\ n-(r-2) \end{array}\right)10^{\left(r-2)(\Theta +1\right)}+\cdots +1\;\mathrm{mod}\;10^{r(\Theta +1)}$$

by Lemma 1, we have

$$\left(\begin{array}{c} n\\ n-(r-1) \end{array}\right)10^{\left(r-1)(\Theta +1\right)}+\left(\begin{array}{c} n\\ n-(r-2) \end{array}\right)10^{\left(r-2)(\Theta +1\right)}+\cdots +1<10^{r(\Theta +1)}$$

therefore,

$${\left(10^{\Theta +1}+1\right)}^{n}\;\text{mod}\;10^{r(\Theta +1)}=\left(\begin{array}{c} n\\ n-(r-1) \end{array}\right)10^{\left(r-1)(\Theta +1\right)}+\left(\begin{array}{c} n\\ n-(r-2) \end{array}\right)10^{\left(r-2)(\Theta +1\right)}+\cdots +1$$

 □

Corollary 1 *The integer* $\left(\begin{array}{c} n\\ n-(r-1) \end{array}\right)10^{\left(r-1)(\Theta +1\right)}+\left(\begin{array}{c} n\\ n-(r-2) \end{array}\right)10^{\left(r-2)(\Theta +1\right)}+\cdots +1$ *has at most* r(Θ+1) *significant digits.*

Proof This follows directly from how

$$\left(\begin{array}{c} n\\ n-(r-1) \end{array}\right)10^{\left(r-1)(\Theta +1\right)}+\left(\begin{array}{c} n\\ n-(r-2) \end{array}\right)10^{\left(r-2)(\Theta +1\right)}+\cdots +1$$

is the remainder when ${\left(10^{\left(\Theta +1\right)}+1\right)}^{n}$ mod $10^{r(\Theta +1)}$. □

Corollary 2 *The left most partition of length* (Θ+1) *from the right of the integer*

$$\left(\begin{array}{c} n\\ n-(r-1) \end{array}\right)10^{\left(r-1)(\Theta +1\right)}+\left(\begin{array}{c} n\\ n-(r-2) \end{array}\right)10^{\left(r-2)(\Theta +1\right)}+\cdots +1\;\text{is}\;\left(\begin{array}{c} n\\ n-(r-1) \end{array}\right)=\left(\begin{array}{c} n\\ r-1 \end{array}\right).$$

Proof By Corollary 1, the integer

$$\left(\begin{array}{c} n\\ n-(r-1) \end{array}\right)10^{\left(r-1)(\Theta +1\right)}+\left(\begin{array}{c} n\\ n-(r-2) \end{array}\right)10^{\left(r-2)(\Theta +1\right)}+\cdots +1$$

has at most r(Θ+1) significant digits, and similarly

$$\left(\begin{array}{c} n\\ n-(r-2) \end{array}\right)10^{\left(r-2)(\Theta +1\right)}+\left(\begin{array}{c} n\\ n-(r-3) \end{array}\right)10^{\left(r-3)(\Theta +1\right)}+\cdots +1$$

has at most (r−1)(Θ+1) significant digits. Since $\left(\begin{array}{c} n\\ n-(r-1) \end{array}\right)10^{\left(r-1)(\Theta +1\right)}$ has (r−1)(Θ+1) zeros to the right and it has at most r(Θ+1) significant digits, the left most partition of length Θ+1 of $\left(\begin{array}{c} n\\ n-(r-1) \end{array}\right)10^{\left(r-1)(\Theta +1\right)}$ and $\left(\begin{array}{c} n\\ n-(r-1) \end{array}\right)10^{\left(r-1)(\Theta +1\right)}+\left(\begin{array}{c} n\\ n-(r-2) \end{array}\right)10^{\left(r-2)(\Theta +1\right)}+\cdots +1$ are the same.

Since the left most partition of $\left(\begin{array}{c} n\\ n-(r-1) \end{array}\right)10^{\left(r-1)(\Theta +1\right)}$ is

$$\left(\begin{array}{c} n\\ n-(r-1) \end{array}\right)=\left(\begin{array}{c} n\\ r-1 \end{array}\right),$$

the left most partition of

$$\left(\begin{array}{c} n\\ n-(r-1) \end{array}\right)10^{\left(r-1)(\Theta +1\right)}+\left(\begin{array}{c} n\\ n-(r-2) \end{array}\right)10^{\left(r-2)(\Theta +1\right)}+\cdots +1\;\text{is}\;\left(\begin{array}{c} n\\ r-1 \end{array}\right).$$

 □

Theorem 1 *The* $r^{\text{th}}$ *partition of* (Θ+1) *digits from the right of the integer* ${\left(1\underset{\Theta \;\text{zeros}}{\underset{︸}{0\cdots 0}}1\right)}^{n}$ *is the binomial coefficient* $\left(\begin{array}{c} n\\ r-1 \end{array}\right)$ *, where* $\Theta =\lfloor \log_{10}\left(\begin{array}{c} n\\ \left\lfloor \frac{n}{2}\right\rfloor \end{array}\right)\rfloor$ *.*

Proof The $r^{\text{th}}$ partition of (Θ+1) digits from the right of the integer ${\left(1\underset{\text{Θ zeros}}{\underset{︸}{0\cdots 0}}1\right)}^{n}={\left(10^{\Theta +1}+1\right)}^{n}$ is the left most partition of ${\left(1\underset{\text{Θ zeros}}{\underset{︸}{0\cdots 0}}1\right)}^{n}\;\text{mod}\;10^{r\times (\Theta +1)}$.

From Proposition 1,

$${\left(1\underset{\text{Θ zeros}}{\underset{︸}{0\cdots 0}}1\right)}^{n}\;\text{mod}\;10^{r\times (\Theta +1)}=\left(\begin{array}{c} n\\ n-(r-1) \end{array}\right)10^{\left(r-1)(\Theta +1\right)}+\left(\begin{array}{c} n\\ n-(r-2) \end{array}\right)10^{\left(r-2)(\Theta +1\right)}+\cdots +1$$

Again from Corollary 2, the left most (Θ+1) many digits of

$$\left(\begin{array}{c} n\\ n-(r-1) \end{array}\right)10^{\left(r-1)(\Theta +1\right)}+\left(\begin{array}{c} n\\ n-(r-2) \end{array}\right)10^{\left(r-2)(\Theta +1\right)}+\cdots +1\;\text{is}\;\left(\begin{array}{c} n\\ r-1 \end{array}\right).$$

 □

Hence, the Θ+1 digits partition from the right of the digits of the integer ${\left(1\underset{\text{Θ zeros}}{\underset{︸}{0\cdots 0}}1\right)}^{n}$ generates all the binomial coefficients or the numbers in the cells of the $n^{\text{th}}$ row of the Pascal's triangle.

## 4 Conclusion

Sir Isaac Newton hinted that binomial coefficients in the $n^{\text{th}}$ row of the Pascal's triangle may be achieved from partitioning the digits in the $n^{\text{th}}$ power of some number that contains 11 in some form [25]. It has been shown earlier that the weighted sum of the values in the $n^{\text{th}}$ row of the Pascal's triangle is ${\left(11\right)}^{n}$ [26]. We have proved that (Θ+1) digit partitions of the digits of ${\left(1\underset{\text{Θ zeros}}{\underset{︸}{0\cdots 0}}1\right)}^{n}$ from the right give the values of the cells in the $n^{\text{th}}$ row of the Pascal's triangle, and provided an explicit formula for the value of Θ as a function only of n.

## Declarations

### Author contribution statement

Md. Shariful Islam: Performed the experiments; Contributed reagents, materials, analysis tools or data.

Md. Robiul Islam: Conceived and designed the experiments; Performed the experiments; Analyzed and interpreted the data; Wrote the paper.

Md. Shorif Hossan: Conceived and designed the experiments; Analyzed and interpreted the data; Contributed reagents, materials, analysis tools or data; Wrote the paper.

Md. Hasan Kibria: Performed the experiments.

### Funding statement

This research did not receive any specific grant from funding agencies in the public, commercial, or not-for-profit sectors.

### Data availability statement

No data was used for the research described in the article.

### Declaration of interests statement

The authors declare no conflict of interest.

### Additional information

No additional information is available for this paper.
